# Chlorhexidine is not effective at any concentration in preventing ventilator-associated pneumonia: a systematic review and network meta-analysis

**DOI:** 10.1186/s44158-024-00166-2

**Published:** 2024-05-03

**Authors:** Alessandro De Cassai, Tommaso Pettenuzzo, Veronica Busetto, Christian Legnaro, Chiara Pretto, Alessio Rotondi, Annalisa Boscolo, Nicolò Sella, Marina Munari, Paolo Navalesi

**Affiliations:** 1 Sant’Antonio Anesthesia and Intensive Care Unit, University Hospital of Padua, Padua, Italy; 2 UOC Anesthesia and Intensive Care Unit, University Hospital of Padova, Padua, Italy; 3 Cardiac Surgery Intensive Care Unit, University Hospital of Padua, Padua, Italy; 4 Department of Medicine - DIMED, University of Padova, Padua, Italy; 5 Thoracic Surgery and Lung Transplant Unit - Department of Cardiac, Thoracic, Vascular Sciences, and Public Health, University of Padua, Padua, Italy

**Keywords:** Critical care, Chlorhexidine, Meta-analysis, Ventilator-associated pneumonia, Mechanical ventilation

## Abstract

**Introduction:**

Oral chlorhexidine has been widely used for ventilator-associated pneumonia prevention in the critical care setting; however, previous studies and evidence synthesis have generated inconsistent findings. Our study aims to investigate if different concentrations of oral chlorhexidine may be effective in preventing such complication in intensive care unit patients.

**Methods:**

After pre-registration (Open Science Framework: 8CUKF), we conducted a network meta-analysis with the following PICOS: adult patients (age > 18 years old) undergoing invasive mechanical ventilation admitted in ICU (P); any concentration of chlorhexidine used for oral hygiene (I); placebo, sham intervention, usual care, or no intervention (C); rate of VAP (primary outcome), mechanical ventilation length, ICU length of stay (LOS), hospital LOS, mortality (secondary outcomes) (O); randomized controlled trials (S). We used the following database: PubMed, the Cochrane Central Register of Controlled Trials (CENTRAL), Scopus, and EMBASE without any limitation in publication date or language.

**Results:**

Chlorhexidine did not demonstrate any significant advantage over the control group in preventing ventilator-associated pneumonia or reducing mortality, duration of mechanical ventilation, length of stay in the intensive care unit, or overall mortality.

**Conclusions:**

Chlorhexidine oral decontamination does not reduce the rate of ventilator-associated pneumonia in critically ill adult patients and its routine use could not be recommended.

**Trial registration:**

Registration number: Open Science Framework: 8CUKF.

**Supplementary Information:**

The online version contains supplementary material available at 10.1186/s44158-024-00166-2.

## Introduction

Hospital-acquired infections contribute to prolonging hospital stays, increasing patients’ morbidity and mortality, and inflating hospitalization costs [1, 2]. Patients admitted to intensive care units (ICUs) face an increased risk of acquiring such an infection that in some studies has been estimated to be around 30% [3].

Lower respiratory system is the most common site of infection in ICU patients [4].

Ventilator-associated pneumonia (VAP) is a distinct form of pneumonia occurring in patients undergoing invasive mechanical ventilation. Micro-organisms access the respiratory system through entry points, such as the endotracheal tube, or via leakage of secretions around the endotracheal cuff [5]. Numerous factors contribute to the development of VAP in critically ill patients, e.g., the aspiration of gastrointestinal microbes, compromised cough reflex, the inability to effectively clear secretions through the pharynx and mouth, and inadequate oral care [5].

The occurrence of VAP is associated with a mortality risk ranging from 1 to 10% [6]. One of the proposed strategies for VAP prophylaxis is the use of oral chlorhexidine washes to prevent the growth and aspiration of bacteria. Being simple and low-cost, the vast majority of ICUs have adopted daily oral care with chlorhexidine in their patients [7]. Despite the robust rationale, the assessment of oral antiseptics use as a preventive strategy for VAP has generated inconsistent findings in prior studies [8–10]. Moreover, a meta-analysis, overall including 16 randomized controlled trials and 3630 patients, did not support the use of chlorhexidine for the prevention of VAP in non-cardiac surgery patients [11]. However, this work did not consider the effect of the different concentrations of oral chlorhexidine employed in the included studies.

The objective of this network meta-analysis is to assess whether different concentrations of oral chlorhexidine may be effective in preventing VAP in ICU patients. Secondary outcomes were duration of invasive mechanical ventilation, ICU length of stay, and hospital length of stay and mortality.

## Methods

The protocol for this network meta-analysis has been prospectively registered on Open Science Framework [12], and the Preferred Reporting Items for Systematic reviews and Meta-Analysis (PRISMA) statement guidelines was followed for the reporting of the present manuscript [13].

### Eligibility criteria

Studies were considered to be eligible for inclusion using the following PICOS criteria: adult patients (age > 18 years old) undergoing invasive mechanical ventilation admitted in ICU (P); any concentration of chlorhexidine used for oral hygiene (I); placebo, sham intervention, usual care, or no intervention (C); rate of VAP (primary outcome), mechanical ventilation length, ICU length of stay (LOS), hospital LOS, mortality (secondary outcomes) (O); randomized controlled trials (S).

### Search strategy

We performed a systematic search of the medical literature for the identification, screening, and inclusion of articles. We did not apply any restriction related to language or year of publication. We queried the following database from inception to May 17, 2023: PubMed, The Cochrane Central Register of Controlled Trials (CENTRAL), Scopus, and EMBASE.

### Study selection

Three researchers (CL, AS, CP) independently screened titles and abstracts of the identified papers in order to select relevant manuscripts. Each citation was reviewed in full-text form if considered potentially relevant. All the references of the included literature were examined to retrieve further relevant studies. The search strategy for each database is available as supplementary material. After identifying those studies meeting inclusion criteria, two authors (VB, AB) manually reviewed and assessed each of the included studies.

### Quality assessment and certainty of evidence assessment

Risk of bias was assessed independently by two members of the team not previously involved in the study selection phase (ADC, TP). The assessment was performed using the Risk of Bias (RoB) 2 Tool, expressing the overall risk of bias on a three-grade scale (“low risk of bias,” “high risk of bias,” or “some concerns”) [14]. In case of disagreements after discussion among assessors, a third researcher (PN) was consulted.

### Statistical methods

Meta-analysis of data was performed using R version 4.1 (R Foundation for Statistical Computing, Vienna, Austria) and the package “netmeta.” The treatment effect for continuous outcomes was measured using mean difference (MD) with 95% confidence interval (CI). For dichotomous outcomes, we expressed the treatment effect as odds ratio (OR) with 95% CI. Availability of evidence, transitivity assumption, intra-network connectivity, and network coherence were considered to assess the feasibility of conducting a network meta-analysis [15]. To rank comparators, we conducted a ranking analysis using the frequentist analogue of the surface under the cumulative ranking curve (SUCRA) [16]. In case of data expressed as median and quartiles, we utilized Hozo’s method [17] to estimate the mean and standard deviation (SD). Additionally, we abstained from applying continuity correction to cases with zero events.

### Sensitivity analysis

We decided to perform the following post hoc sensitivity analysis for the primary outcome: (a) excluding postoperative patients, (b) excluding high risk-of-bias studies, (c) excluding all comparators other than placebo/no intervention.

### Heterogeneity and publication bias analysis

For assessment of study inconsistency and heterogeneity, the *I*^2^ and Tau^2^ statistics were used. Values of *I*^2^ were categorized as follows: low heterogeneity: *I*^2^ < 25%, moderate heterogeneity: *I*^2^ 25 to 50%, or high heterogeneity: *I*^2^ > 50%) [18]. A random-effect model was preferred, regardless of heterogeneity. Publication bias was evaluated both by visual inspection of funnel plots or Egger’s test when more than ten studies were available for a specific outcome.

## Results

### Study characteristics

**Fig. 1:**
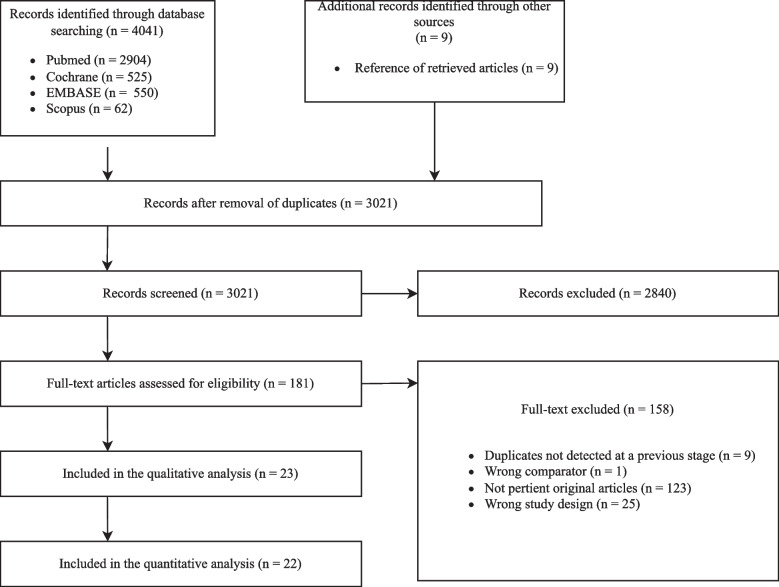
PRISMA flowchart

PRISMA flowchart of the included studies is depicted in Fig. 1. We selected 181 articles for full-text assessment. Of these, only 23 articles met our inclusion criteria [8–10, 19–38]. However, one study was excluded, as it presented a mixed intubated/not intubated cohort of patients, and the corresponding author was unable to provide us with the subset of intubated patients only [38]. Therefore, 22 articles (5314 patients) were eventually included for qualitative and quantitative analysis.

Characteristics of the included studies are reported in Table 1. The identified chlorhexidine concentrations were as follows: 0.12% (nine studies) [8, 20, 22, 24–28, 33], 0.2% (eight studies) [9, 10, 21, 23, 29, 30, 36, 37], 2% (six studies) [19, 30–32, 34, 35]. Among these studies, only one performed a direct comparison among chlorhexidine concentrations, i.e., 0.2% vs 2.0% [30].

**Table 1:** Study characteristics

Study	Country	Population	Main outcome	Group 1	Group 2	Nt	VAP criteria
Meinberg (2012) [19]	Brazil	ICU	VAP	Chlorhexidine 2%	Placebo	4	CDC
Scannapieco (2009) [20]	USA	ICU	Oral bacterial colonization	Chlorhexidine 0.12%	Placebo	2	CPIS or PC
Ozczka (2012) [21]	Turkey	ICU	VAP	Chlorhexidine 2%	Placebo	4	PC
De Riso (1996) [22]	USA	Postsurgical	Nosocomial infections	Chlorhexidine 0.12%	Placebo	2	CDC
Fourrier (2005) [9]	France	ICU	Nosocomial infections	Chlorhexidine 0.2%	Placebo	≥ 3	CDC
Segers (2006) [8]	The Netherlands	Postsurgical	Nosocomial infections	Chlorhexidine 0.12%	Placebo	4	CDC
Panchabhai (2009) [23]	India	ICU	VAP	Chlorhexidine 0.2%	Sham (potassium permanganate)	2	CDC
Bellissimo-Rodrigues (2009)[24]	Brazil	ICU	Nosocomial respiratory infections	Chlorhexidine 0.12%	Placebo	3	CDC
Dale (2021) [25]	Canada	ICU	ICU Mortality	Chlorhexidine 0.12%	Usual care	3	CDC
Dale (2009) [26]	USA	ICU	VAP	Chlorhexidine 0.12%	Usual care	3	CPIS
Zarinfar (2021) [27]	Iran	ICU	VAP	Chlorhexidine 0.12%	Usual care	2	CPIS and PC
Jo Grap (2011) [28]	USA	Trauma	VAP	Chlorhexidine 0.12%	Usual care	1	CPIS
Jahanshir (2022) [29]	Iran	ICU	VAP	Chlorhexidine 0.2%	Sham (clove extract)	2	mCPIS
Zand (2017) [30]	Iran	ICU	VAP	Chlorhexidine 0.2%	Chlorhexidine	1	CPIS
Lin (2015) [31]	China	Postsurgical	VAP	Chlorhexidine 0.2%	Normal saline	3	CPIS
Tantipong (2008) [10]	Thailand	ICU	VAP	Chlorhexidine 2.0%	Normal saline	4	CDC
Tuon (2016) [32]	Brazil	ICU	VAP	Chlorhexidine 2.0%	Normal saline	2	CDC
Pobo (2009) [33]	Spain	ICU	VAP	Chlorhexidine 0.12%	Usual care	3	CDC
Meidani (2018) [34]	Iran	ICU	VAP	Chlorhexidine 0.2%	Placebo	2	CDC
Koeman (2006) [35]	The Netherlands	ICU	VAP	Chlorhexidine 2%	Placebo	4	Clinical decision
Fourrier (2000) [36]	France	ICU	Oral bacterial colonization	Chlorhexidine 0.2%	Sham (bicarbonate)	3	CDC
Berry (2009) [37]	Australia	ICU	Oral bacterial colonization	Chlorhexidine 0.2%	Sham (sterile water)	2	Clinical decision

*ICU* intensive care unit, *CDC* Center for Disease Control and Prevention, *CPIS* clinical pulmonary infection score, *PC* positive culture, *Nt* number of times the treatment was applied

### Risk of bias assessment

**Fig. 2:**
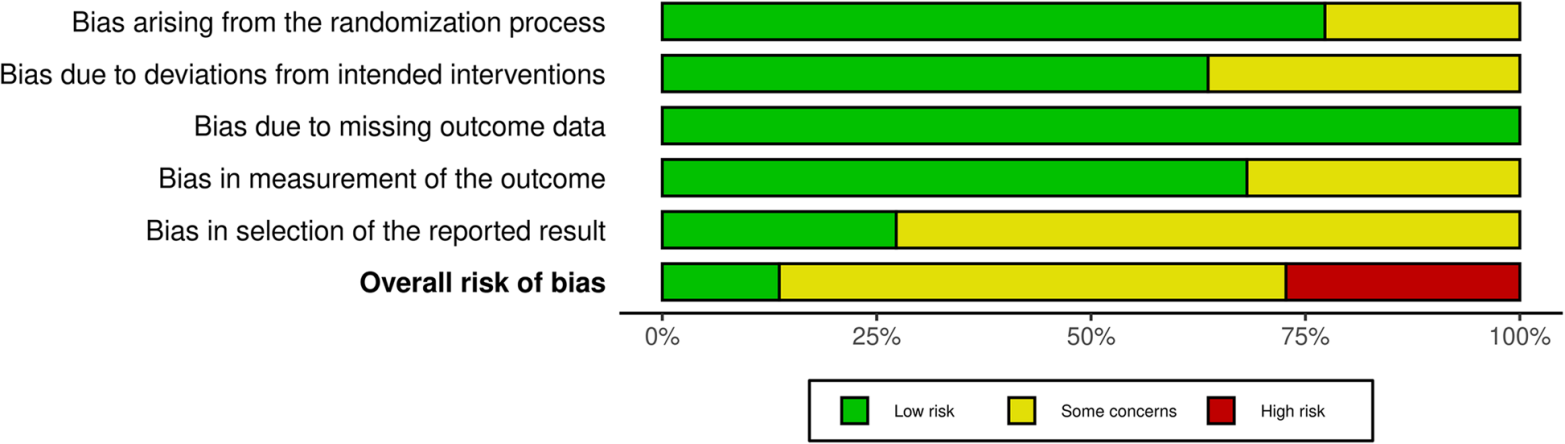
Overall risk of bias assessment

The overall risk of bias assessment is summarized in Fig. 2 and detailed in Additional file 2. Six studies were evaluated to be at high risk of bias, thirteen studies to some concern, while the remaining studies were evaluated to be at low risk of bias.

### Outcomes

Results for all the outcomes are summarized in Table 2, and the SUCRA analysis is shown in Table 3. Publication bias was detected for no outcomes (Additional file 3).

**Table 2:** Study outcomes

Group	VAP (OR, 95% CI)	*p*-value	MV length (MD, 95% CI)	*p*-value	ICU LOS (MD, 95% CI)	*p*-value	Hospital LOS (MD, 95% CI)	*p*-value	Mortality (OR, 95% CI)	*p*-value
0.12%	0.70 (0.41;1.19)	0.195	− 0.25 (− 1.55; 1.04)	0.699	0.46 (− 1.25; 2.17)	0.597	− 0.60 (− 0.72; − 0.47)	< 0.001*	1.02 (0.73–1.42)	0.896
0.2%	0.93 (0.52;1.65)	0.806	0.63 (− 1.02; 2.29)	0.453	− 1.51 (− 3.36; 0.34)	0.110	− 1.64 (− 5.08; 1.79)	0.349	1.16 (0.78; 1.72)	0.439
2.0%	0.56 (0.28;1.13)	0.104	0.22 (− 1.88; 2.32)	0.836	0.11 (− 2.67; 2.90)	0.935	− 1.53 (− 7.25; 4.19)	0.599	0.64 (0.31; 1.34)	0.241

Placebo, sham intervention, usual care, or no intervention are used as reference group

*CI* confidence intervals, *ICU* intensive care unit, *LOS* length of stay, *MD* mean difference, *MV* mechanical ventilation, *OR* odds ratio, *VAP* ventilator-associated pneumonia

^*^Statistically significant

**Table 3:** Surface under the cumulative ranking curve analysis

Group	VAP	Mechanical ventilation length	ICU LOS	Hospital LOS	Mortality
Reference	0.184	0.568	0.429	0.158	0.484
0.12%	0.320	0.698	0.260	0.550	0.424
0.2%	0.654	0.270	0.903	0.687	0.196
2.0%	0.842	0.463	0.406	0.603	0.895

Placebo, sham intervention, usual care, or no intervention are used as the reference group

*ICU* intensive care unit, *LOS* length of stay, *VAP* ventilator-associated pneumonia

#### VAP

Twenty-one studies evaluated VAP in 6626 patients overall (367 patients assigned to the 2.0% concentration, 464 to the 0.2%, 2471 to the 0.12%, and 3324 assigned to the control group). None of the concentrations of chlorhexidine was associated with a statistically significant reduction in VAP, when compared to either non-chlorhexidine comparators or other chlorhexidine concentrations (Table 1). Heterogeneity was high (*I*^2^ 66.8%, Tau^2^ 0.39). The estimation of direct and indirect evidence is available as supplementary material (Additional file 4), while the graph describing the network among intervention is presented as Additional file 5. There was no evidence of publication bias at the funnel plot (Egger test *p*-value 0.635).

### Mechanical ventilation duration

Twelve studies evaluated the mechanical ventilation duration and randomized 5379 patients (255 to the 2.0% concentration, 188 to the 0.2%, 2191 to the 0.12%, while 2745 were assigned to the control group). The utilization of chlorhexidine at any concentration was not found to reduce mechanical ventilation duration, in comparison with the control group. Heterogeneity was high (*I*^2^ 56%; Tau^2^ 1.23). No publication was detected at funnel plot (*p*-value 0.456).

### ICU LOS

Ten studies reported results for ICU LOS, randomizing a total of 2040 patients (255 to the 2.0% concentration, 188 to the 0.2%, 594 to the 0.12%, and 1003 to the control group). We did not observe shorter ICU LOS in patients receiving any concentration of chlorhexidine, when compared to the control group. Heterogeneity was low (*I*^2^ 21.7%, Tau^2^ 1.02), and there were no signs of publication bias (*p*-value 0.457).

### Hospital LOS

Analysis of seven studies, randomizing a total of 1834 patients (155 to the 2.0% concentration, 70 to the 0.2%, 695 to the 0.12%, while 914 to the control group), found only a statistically significant but clinically unimportant reduced LOS in 0.12% patients (− 0.60; CI − 0.72; − 0.47 days). In this analysis, there was low heterogeneity (*I*^2^ 0%, Tau^2^ 0) without publication bias at the visual inspection of the funnel plot.

### Mortality

Fourteen studies reported data for this outcome, enrolling 5978 patients overall (185 in the 2.0% group, 347 in the 0.2% group, 2447 in the 0.12% group, while 2999 patients in the control group). No significant effect was detected for any of the intervention. Low heterogeneity was reported (*I*^2^ 21.4%, Tau^2^ 0.045) and no publication bias (*p*-value 0.490).

### Sensitivity analysis

Results for the sensitivity analyses are reported in Table 4. Briefly, none of the subgroup analyses was able to determine the superiority of chlorhexidine at any concentration over controls.

**Table 4:** Sensitivity analysis for the primary outcome

	Risk of bias			No postoperative patients			Only placebo/usual care		
	*N* (6138)	OR (95% CI)	*p*-value	*N* (5467)	OR (95% CI)	*p*-value	*N* (6469)	OR (95% CI)	*p*-value
Control	3078	Ref	Ref	2751	Ref	Ref	3250	Ref	Ref
0.12%	2590	0.70 (0.44; 1.12)	0.140	1932	0.81 (0.41; 1.61)	0.557	2590	0.70 (0.44; 1.11)	0.132
0.2%	211	1.42 (0.71; 2.79)	0.317	464	0.94 (0.50; 1.77)	0.858	262	0.97 (0.49; 1.91)	0.935
2.0%	259	0.57 (0.27; 1.21)	0.145	320	0.64 (0.28; 1.47)	0.293	367	0.55 (0.30; 1.05)	0.069
*I*^2^	62.6			70.5			56.5		
Tau^2^	0.27			0.52			0.25		

*CI* confidence interval, *N* number of patients, *OR* odds ratio

## Discussion

This systematic review and network meta-analysis including 5314 adult patients from 21 RCTs found that no concentration of chlorhexidine was associated with reduced rate of VAP, in comparison either with other chlorhexidine concentrations or with no-chlorhexidine interventions. Moreover, compared to other chlorhexidine concentrations or no-chlorhexidine interventions, no concentration of chlorhexidine improved mechanical ventilation duration, LOS, and mortality.

Since the finding is that no concentration of chlorhexidine is effective in preventing VAP or any of the other investigated outcomes, our network meta-analysis contributes additional evidence to a previous pairwise meta-analysis, published in 2014 [11], already questioning the impact of chlorhexidine on preventing VAP in non-cardiac surgery patients.

Previous meta-analysis [39, 40] showed that certain mode of chlorhexidine delivery (solution but no gel or rinse) or frequency of use (4 times/die) could have an impact on VAP incidence; however, the paucity of mode of deliveries and frequency of administrations when subcategorized for chlorhexidine solutions prevented us to conduct such subgroups analysis. This remains for sure an interesting point for future research.

Moreover, 0.12% chlorhexidine concentration group is more represented (2590 patients), compared to the 0.2% (464 patients) and 2% (367 patients) groups. Therefore, expanding the sample size for the other groups might reveal significant benefits on the rate of VAP. However, higher chlorhexidine concentrations may increase the risk of oral lesions [40], selecting the growth of germs resistant to chlorhexidine [41, 42].

Over the years, there have been significant changes in guidelines regarding the use of chlorhexidine for preventing VAP in ventilated patients.

When examining the guidelines, it is essential to note that the “Zero-VAP” bundle (Spanish guidelines) [43] suggested the standard use of chlorhexidine to prevent VAP, recommending concentrations as high as 2%. However, not all the scientific societies agreed with such a recommendation. In fact, in the same year (2014), the SHEA/IDSA guidelines [44] categorized oral care with chlorhexidine under special approaches instead of basic practices due to potential risks and unclear benefits.

A more recent European guideline [45] does not provide a formal recommendation on the use of chlorhexidine for oral care in mechanically ventilated patients due to a lack of safety data and an unclear balance between the potential reduction in VAP and the potential increase in mortality. The latest update from SHEA [46] does not recommend the use of oral chlorhexidine as it may increase mortality rates. Our study aligns with the most recent guidelines and further strengthened these recommendations.

Our research has some limitations that warrants discussion. First, intransitivity may have arisen from the inclusion of studies published over a 26-year period. Over this period, there could have been substantial modifications to VAP prevention bundles, antimicrobial therapies, and other clinical practices, potentially impacting the research outcomes. Second, the main analysis on primary outcome showed high heterogeneity that was not explained by our subgroup analyses, reducing the overall confidence in our results. The among-studies heterogeneity in protocols for chlorhexidine oral decontamination and antimicrobial stewardship and outcome definitions may explain such finding. Third, we included all non-chlorhexidine interventions, i.e., placebo, sham intervention, usual care, and no intervention, in the same group.

## Conclusion

Chlorhexidine oral decontamination does not reduce the rate of VAP in critically ill adult patients in the ICU, and we could not recommend its routine use. Nevertheless, further research is warranted, particularly investigating the potential benefits of chlorhexidine at higher concentrations.

### Supplementary Information

**Additional file 1.** Search strategy.

**Additional file 2.** Risk of bias assessment.

**Additional file 3.** Funnel plots.

**Additional file 4.** Direct–indirect evidence for the main outcome.

**Additional file 5.** Network graph.

## Data Availability

No datasets were generated or analysed during the current study.

## Abbreviations

ICU: Intensive care unit
LOS: Length of stay
MD: Mean difference
OR: Odds ratio
SD: Standard deviation
SUCRA: Surface under the cumulative ranking curve
VAP: Ventilator-associated pneumonia
