# Supplementary material for: Chlorhexidine is not effective at any concentration in preventing ventilator-associated pneumonia: a systematic review and network meta-analysis
Source: J Anesth Analg Crit Care. 2024 May 3;4:30. doi: 10.1186/s44158-024-00166-2 (PMC11067293; doi:10.1186/s44158-024-00166-2)
Supplement: Supplementary file 1 — Additional file 1. Search strategy. [file 44158_2024_166_MOESM1_ESM.docx]

**Supplementary Material 1 – Search strategy**

| **Pubmed** |
| --- |
| (("CRITICAL ILLNESS") OR ("intensive care" or intensive‐care or "critical care" or critical‐care)) AND (VAP OR pneumonia OR nosocomial infection OR "ventilator associated") AND (oral OR dental OR mouthwashes OR chlorhexidine OR toothbrush OR Corsodyl OR Peridex OR Hibident or Prexidine or Parodex or Chlorexil or Peridont or Eludril or Perioxidin or Chlorohex or Savacol or Periogard or Chlorhexamed or Nolvasan or Sebidin or Tubulicid or hibitane)) |
| **Cochrane** |
| (("CRITICAL ILLNESS") OR ("intensive care" or intensive‐care or "critical care" or critical‐care)) AND (VAP OR pneumonia OR nosocomial infection OR "ventilator associated") AND (oral OR dental OR mouthwashes OR chlorhexidine OR toothbrush OR Corsodyl OR Peridex OR Hibident or Prexidine or Parodex or Chlorexil or Peridont or Eludril or Perioxidin or Chlorohex or Savacol or Periogard or Chlorhexamed or Nolvasan or Sebidin or Tubulicid or hibitane)) |
| **EMBASE** |
| ((Intubated or intubation) and (oral or hygiene or toothbrush or mouthwashes or chlorexidine)).mp. and (ventilation or infection).ti. [mp=title, abstract, heading word, drug trade name, original title, device manufacturer, drug manufacturer, device trade name, keyword heading word, floating subheading word, candidate term word] |
| **Scopus** |
| "critical care" AND pneumonia AND chlorhexidine |
