# Supplementary material for: Chlorhexidine is not effective at any concentration in preventing ventilator-associated pneumonia: a systematic review and network meta-analysis
Source: J Anesth Analg Crit Care. 2024 May 3;4:30. doi: 10.1186/s44158-024-00166-2 (PMC11067293; doi:10.1186/s44158-024-00166-2)
Supplement: Supplementary file 2 — Additional file 2. Risk of bias assessment. [file 44158_2024_166_MOESM2_ESM.docx]

**Supplementary Digital Content 2 - Risk of Bias**

1)Meinberg (2012)

|  |  |
| --- | --- |
| D1 | A random component was used in the sequence generation process. No imbalances are apparent. |
| D2 | Personnel and patients were not aware of intervention. Appropriate analysis to estimate the effect of assignment. |
| D3 | Outcome data for all participants |
| D4 | Outcome assessor was probably blinded to the intervention |
| D5 | Trial analysis was not performed according to a prespecified plan |
| Overall | Some concerns |

2)Scannapieco (2009)

|  |  |
| --- | --- |
| D1 | A random component was used in the sequence generation process. No imbalances are apparent. |
| D2 | Personnel and patients were not aware of intervention. Appropriate analysis to estimate the effect of assignment. |
| D3 | Outcome data for all participants |
| D4 | Outcome assessor was blinded to the intervention |
| D5 | Trial analysis was performed according to a prespecified plan (NCT00123123) |
| Overall | Low risk |

3)Ozczka (2012)

|  |  |
| --- | --- |
| D1 | A random component was used in the sequence generation process. No imbalances are apparent. |
| D2 | Personnel and patients were not aware of intervention. Appropriate analysis to estimate the effect of assignment. |
| D3 | Outcome data for all participants |
| D4 | Outcome assessor was blinded to the intervention |
| D5 | Trial analysis was not performed according to a prespecified plan |
| Overall | Some concerns |

4)De Riso (1996)

|  |  |
| --- | --- |
| D1 | A random component was used in the sequence generation process. No imbalances are apparent. |
| D2 | Personnel and patients were not aware of intervention. Appropriate analysis to estimate the effect of assignment. |
| D3 | Outcome data for all participants |
| D4 | Outcome assessor was probably blinded to the intervention |
| D5 | Trial analysis was not performed according to a prespecified plan |
| Overall | Some concerns |

5)Fourrier (2005)

|  |  |
| --- | --- |
| D1 | A random component was used in the sequence generation process. No imbalances are apparent. |
| D2 | Personnel and patients were not aware of intervention. Appropriate analysis to estimate the effect of assignment. |
| D3 | Outcome data not for all participants, but no evidence that results are biased |
| D4 | Outcome assessor was blinded to the intervention |
| D5 | Trial analysis was not performed according to a prespecified plan |
| Overall | Some concerns |

6) Segers (2006)

|  |  |
| --- | --- |
| D1 | A random component was used in the sequence generation process. No imbalances are apparent. |
| D2 | Personnel were probably aware of intervention. Appropriate analysis to estimate the effect of assignment. |
| D3 | Outcome data for all participants |
| D4 | Outcome assessor was blinded to the intervention |
| D5 | Trial analysis was performed according to a prespecified plan (NCT00272675) |
| Overall | Some concerns |

7) Panchabhui (2009)

|  |  |
| --- | --- |
| D1 | A random component was used in the sequence generation process. No imbalances are apparent. |
| D2 | Personnel were probably aware of intervention. Appropriate analysis to estimate the effect of assignment. |
| D3 | Outcome data for all participants |
| D4 | Outcome assessor was probably not blinded to the intervention |
| D5 | Trial analysis plan was probably not pre registered |
| Overall | High risk |

8) Rodrigues (2009)

|  |  |
| --- | --- |
| D1 | A random component was used in the sequence generation process. No imbalances are apparent. |
| D2 | Personnel probably not aware of intervention. |
| D3 | Outcome data for all participants |
| D4 | Outcome assessor was blinded to the intervention |
| D5 | Trial analysis was not preregistered |
| Overall | Some concerns |

9) Dale (2021)

|  |  |
| --- | --- |
| D1 | A random component was used in the sequence generation process. No imbalances are apparent. |
| D2 | Personnel were probably aware of intervention. Appropriate analysis to estimate the effect of assignment. |
| D3 | Outcome data for all participants |
| D4 | Outcome assessor were probably blinded to the intervention |
| D5 | Trial analysis was preregistered (NCT03382730) |
| Overall | Some concerns |

10) Cindy (2009)

|  |  |
| --- | --- |
| D1 | A random component was used in the sequence generation process. No imbalances are apparent. |
| D2 | Personnel were not aware of intervention. Appropriate analysis to estimate the effect of assignment. |
| D3 | Outcome data for all participants |
| D4 | Outcome assessor were probably blinded to the intervention |
| D5 | Trial analysis was probably not pre registered |
| Overall | Some concerns |

11) Zarinfar (2021)

|  |  |
| --- | --- |
| D1 | A random component was probably used in the sequence generation process. No imbalances are apparent. |
| D2 | Personnel and patients were not probably aware of intervention. Appropriate analysis to estimate the effect of assignment. |
| D3 | Outcome data for all participants |
| D4 | Outcome assessor were probably not blinded to the intervention |
| D5 | Trial analysis was probably not pre registered |
| Overall | Some concerns |

12) Jo Grap (2021)

|  |  |
| --- | --- |
| D1 | A random component was probably used in the sequence generation process. No imbalances are apparent. |
| D2 | Personnel and patients were not probably aware of intervention. Appropriate analysis to estimate the effect of assignment. |
| D3 | Outcome data for all participants |
| D4 | Outcome assessor were probably not blinded to the intervention |
| D5 | Trial analysis was probably not pre registered |
| Overall | Some concerns |

13) Jahanshir (2010)

|  |  |
| --- | --- |
| D1 | A random component was probably used in the sequence generation process. No imbalances are apparent. |
| D2 | Personnel and patients were not aware of intervention. Appropriate analysis to estimate the effect of assignment. |
| D3 | Outcome data for all participants |
| D4 | Outcome assessor was blinded to the intervention |
| D5 | Trial analysis was probably pre registered (Iranian Registry of Clinical Trials |
| Overall | Low risk |

14) Zand (2017)

|  |  |
| --- | --- |
| D1 | A random component was probably used in the sequence generation process. No imbalances are apparent. |
| D2 | Personnel were probably aware of intervention. Appropriate analysis to estimate the effect of assignment. |
| D3 | Outcome data for all participants |
| D4 | Outcome assessor were probably not blinded to the intervention |
| D5 | Trial analysis was pre registered (Iranian Registry of Clinical Trials IRCT2015100624382N1. |
| Overall | Some concerns |

15) Lin (2015)

|  |  |
| --- | --- |
| D1 | A random component was used in the sequence generation process. No imbalances are apparent. |
| D2 | Personnel and patients were not aware of intervention. Appropriate analysis to estimate the effect of assignment. |
| D3 | Outcome data for all participants |
| D4 | Outcome assessor were blinded to the intervention |
| D5 | Trial analysis was pre registered (ChiCTR-TRC-14005125). |
| Overall | Low risk |

16) Tatipong (2008)

|  |  |
| --- | --- |
| D1 | There is no information on concealment . No imbalances are apparent. |
| D2 | Personnel and patients were probably aware of intervention. Appropriate analysis to estimate the effect of assignment. |
| D3 | Outcome data for all participants |
| D4 | Outcome assessor were blinded to the intervention |
| D5 | Trial analysis was probably not pre registered |
| Overall | High risk |

17) Tuon (2016)

|  |  |
| --- | --- |
| D1 | No information on concealment. No imbalances are apparent. |
| D2 | Personnel and patients were not aware of intervention. Appropriate analysis to estimate the effect of assignment. |
| D3 | Outcome data for all participants |
| D4 | Outcome assessor was probably aware of the intervention |
| D5 | Trial analysis was probably not pre registered |
| Overall | High risk |

18) Pobo (2009)

|  |  |
| --- | --- |
| D1 | A random component was used in the sequence generation process. No imbalances are apparent. |
| D2 | Personnel and patients were not aware of intervention. Appropriate analysis to estimate the effect of assignment. |
| D3 | Outcome data for all participants |
| D4 | Outcome assessor were blinded to the intervention |
| D5 | Trial analysis was probably not pre registered . |
| Overall | Some concern |

19) Meidani (2018)

|  |  |
| --- | --- |
| D1 | No information on concealment. No imbalances are apparent. |
| D2 | Personnel and patients were probably aware of intervention. Appropriate analysis to estimate the effect of assignment. |
| D3 | Outcome data for all participants |
| D4 | Outcome assessor were probably not blinded to the intervention |
| D5 | Trial analysis was probably not pre registered . |
| Overall | High risk |

20) Koeman (2006)

|  |  |
| --- | --- |
| D1 | A random component was used in the sequence generation process. No imbalances are apparent. |
| D2 | Personnel and patients were not aware of intervention. Appropriate analysis to estimate the effect of assignment. |
| D3 | Outcome data for all participants |
| D4 | Outcome assessor were probably blinded to the intervention |
| D5 | Trial analysis was probably not pre registered . |
| Overall | Some concern |

21) Fourrier (2000)

|  |  |
| --- | --- |
| D1 | No information on concealment. No imbalances are apparent. |
| D2 | Personnel were probably aware of intervention. Appropriate analysis to estimate the effect of assignment. |
| D3 | Outcome data for all participants |
| D4 | Outcome assessor were probably not blinded to the intervention |
| D5 | Trial analysis was probably not pre registered . |
| Overall | High risk |

22) Berry (2009)

|  |  |
| --- | --- |
| D1 | No information on concealment. No imbalances are apparent. |
| D2 | Personnel were probably aware of intervention. Appropriate analysis to estimate the effect of assignment. |
| D3 | Outcome data for all participants |
| D4 | Outcome assessor were probably blinded to the intervention |
| D5 | Trial analysis was probably not pre registered . |
| Overall | High risk |
