# Supplementary material for: Chlorhexidine is not effective at any concentration in preventing ventilator-associated pneumonia: a systematic review and network meta-analysis
Source: J Anesth Analg Crit Care. 2024 May 3;4:30. doi: 10.1186/s44158-024-00166-2 (PMC11067293; doi:10.1186/s44158-024-00166-2)
Supplement: Supplementary file 3 — Additional file 3. Funnel plots. [file 44158_2024_166_MOESM3_ESM.pdf]

Ventilator Associated Pneumonia

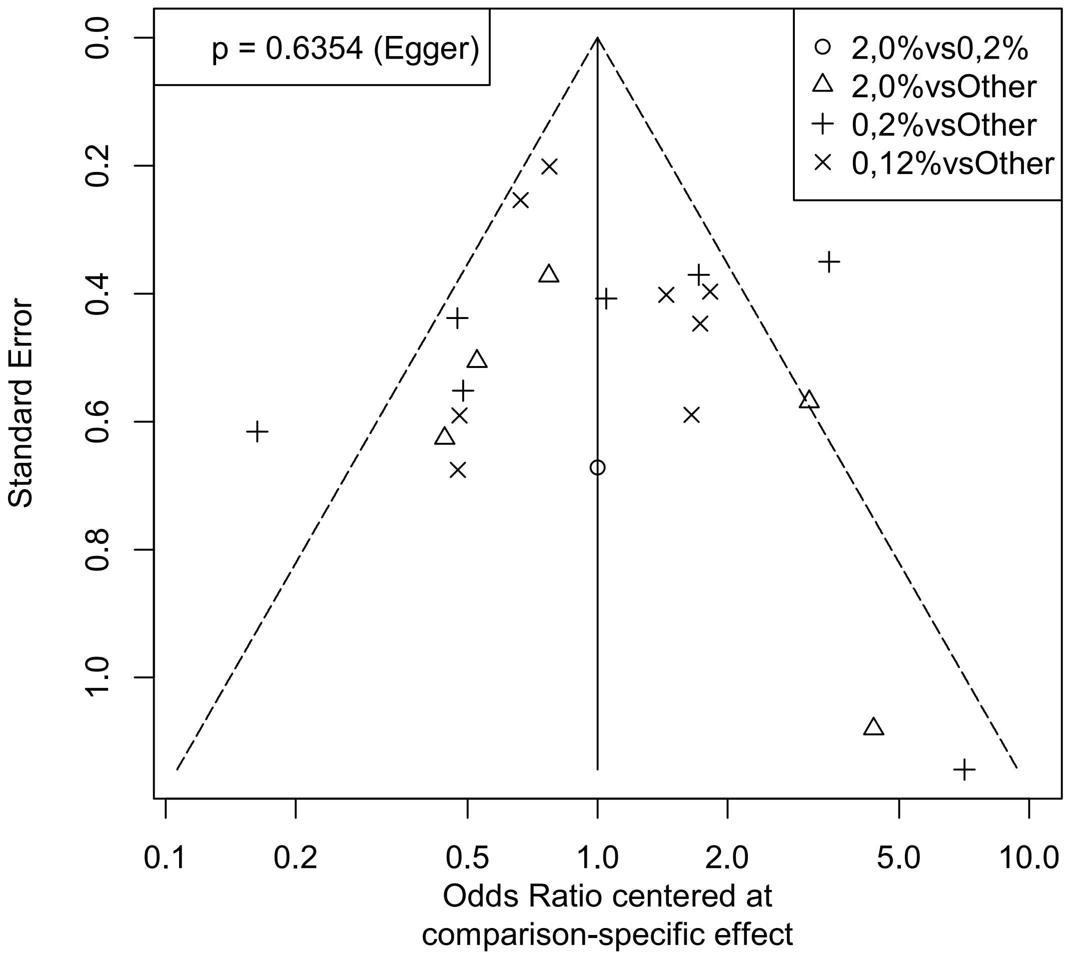

Mechanical Ventilation Lenght

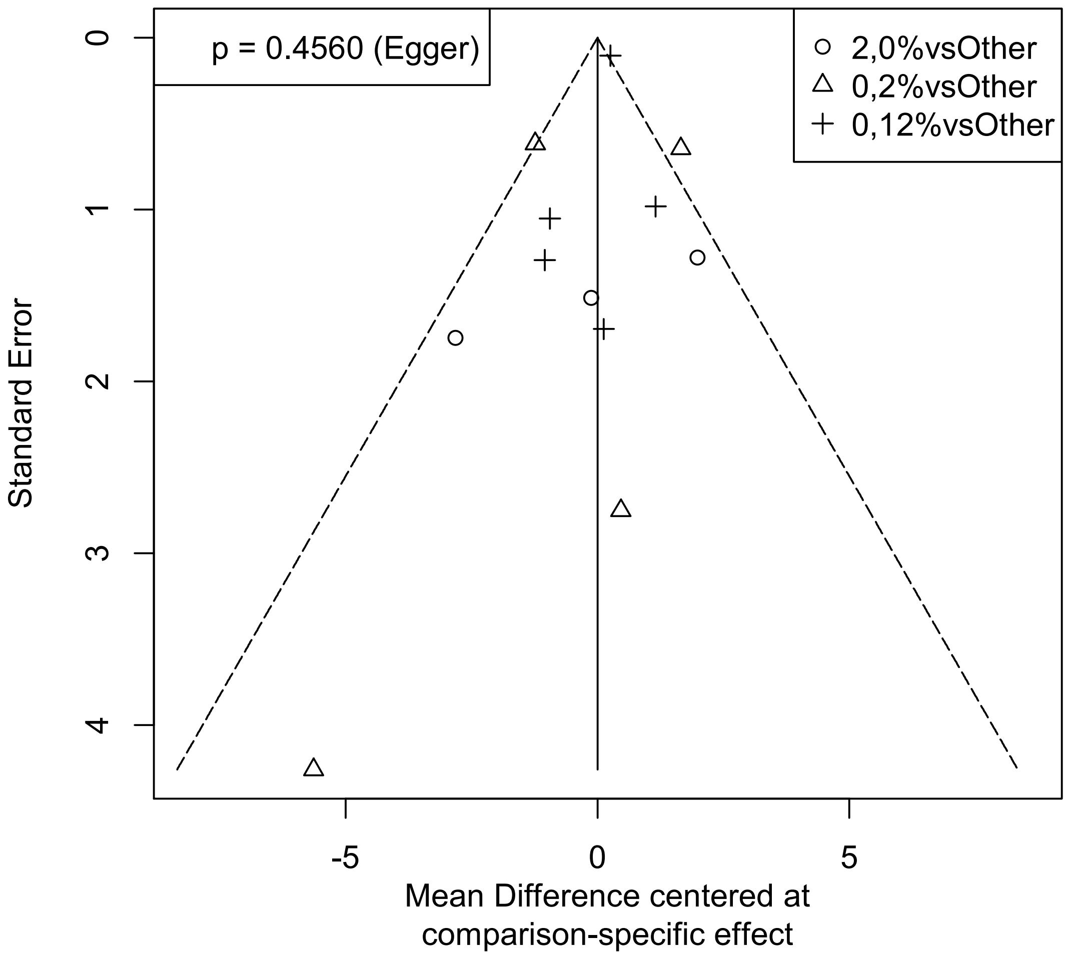

ICU Lenght of Stay

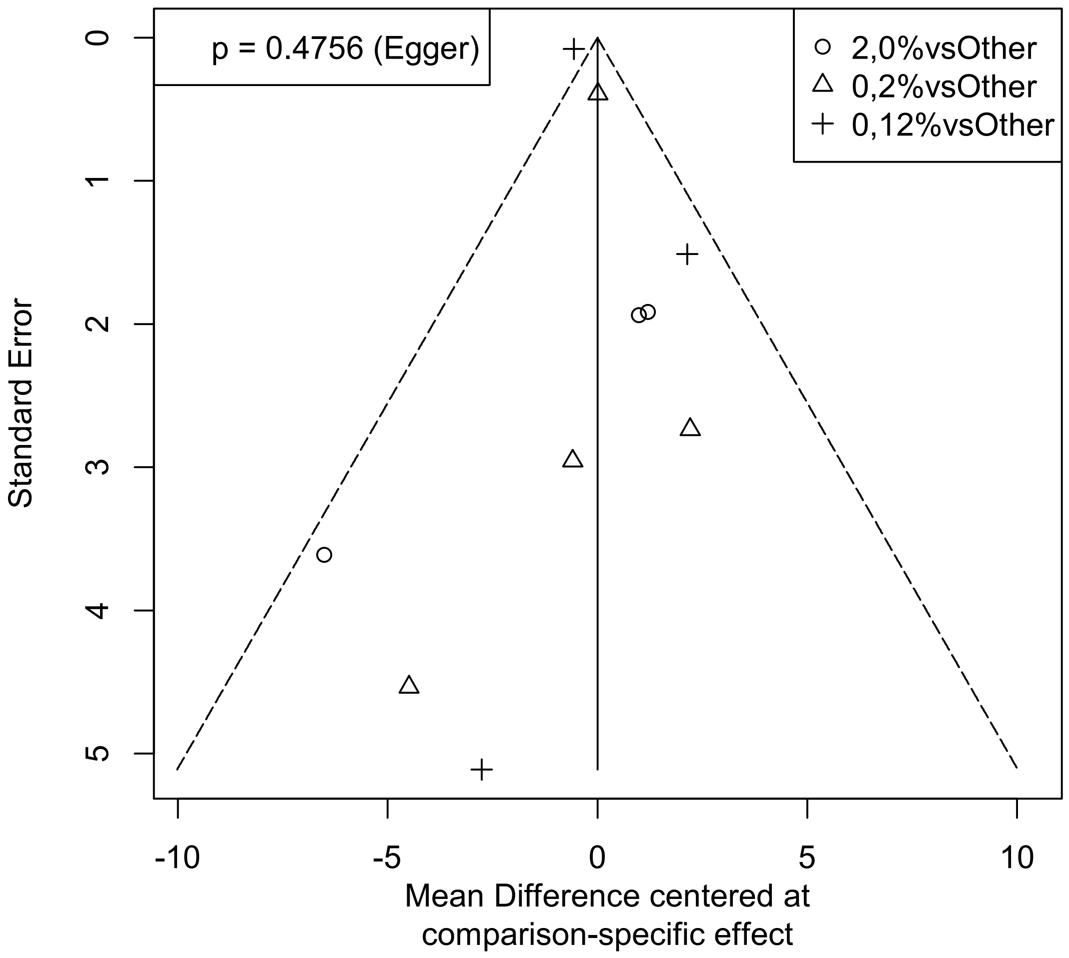

Hospital Lenght of Stay

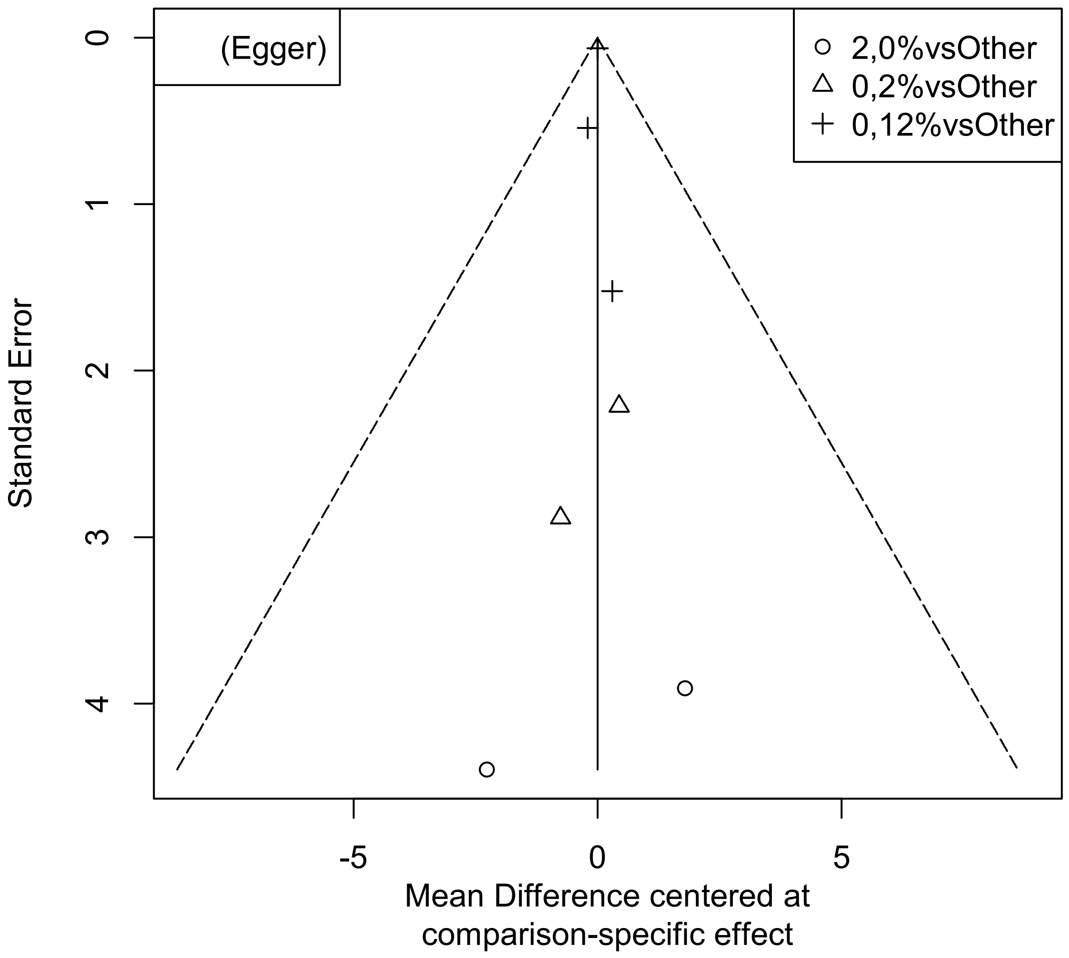

Mortality

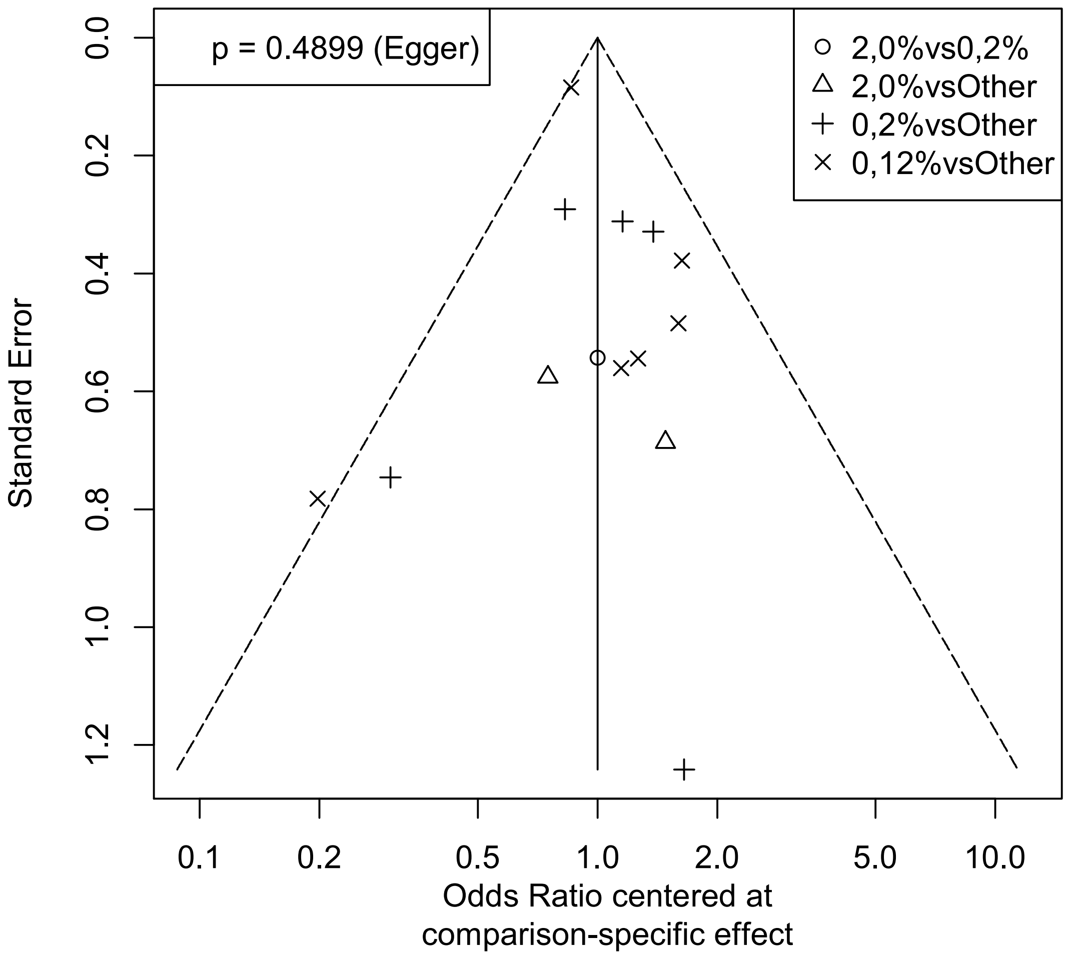

p = 0.4899 (Egger)
2,0%vs0,2%
2,0%vsOther
0,2%vsOther
0,12%vsOther
Standard Error
0.0
0.2
0.4
0.6
0.8
1.0
1.2
0.1
0.2
0.5
1.0
2.0
5.0
10.0
Odds Ratio centered at
comparison-specific effect
