# Supplementary figures and images for: Chlorhexidine is not effective at any concentration in preventing ventilator-associated pneumonia: a systematic review and network meta-analysis
Source: J Anesth Analg Crit Care. 2024 May 3;4:30. doi: 10.1186/s44158-024-00166-2 (PMC11067293; doi:10.1186/s44158-024-00166-2)

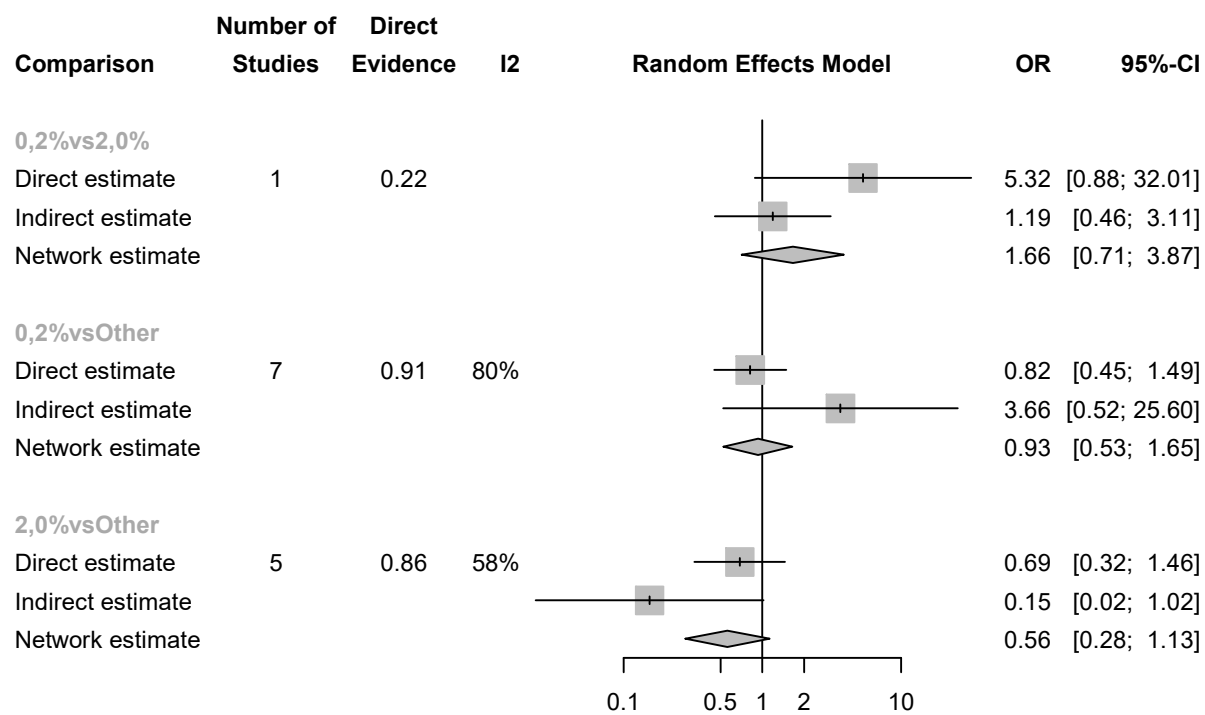

Supplement: Supplementary file 4 — Additional file 4. Direct–indirect evidence for the main outcome. [file 44158_2024_166_MOESM4_ESM.pdf]

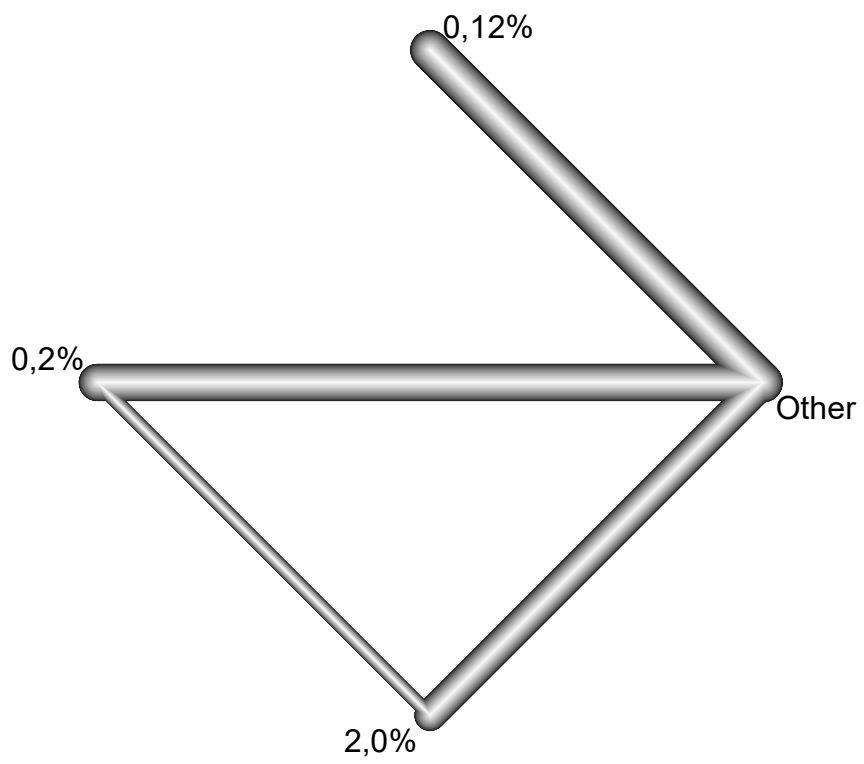

Supplement: Supplementary file 5 — Additional file 5. Network graph. [file 44158_2024_166_MOESM5_ESM.pdf]
